# Peste des Petits Ruminants (PPR) virus serological surveillance in goats in Lao PDR: Issues for disease eradication in a low‐resource disease‐free setting

**DOI:** 10.1111/tbed.13109

**Published:** 2019-01-10

**Authors:** Rebekah J. L. Burns, Bounlom Douangngeun, Watthana Theppangna, Mavuto Mukaka, Matthew D. Wegner, Peter A. Windsor, Stuart D. Blacksell

**Affiliations:** ^1^ Sydney School of Veterinary Science University of Sydney Camden Australia; ^2^ National Animal Health Laboratory Department of Livestock and Fisheries Ministry of Agriculture Vientiane Lao People's Democratic Republic; ^3^ Mahidol‐Oxford Tropical Medicine Research Unit Faculty of Tropical Medicine Mahidol University Bangkok Thailand; ^4^ Centre for Tropical Medicine & Global Health Nuffield Department of Medicine University of Oxford Oxford UK; ^5^ United States Army Medical Directorate ‐ Armed Forces Research Institute of Medical Sciences Bangkok Thailand; ^6^ Lao‐Oxford‐Mahosot Hospital‐Wellcome Trust Research Unit (LOMWRU) Mahosot Hospital Vientiane Lao People's Democratic Republic

**Keywords:** goat, Laos, PPR, serology

## Abstract

Peste des Petits ruminants (PPR) is an economically important transboundary viral disease of goats. This study aimed to determine a baseline of serological evidence for Peste des petits ruminants virus (PPRV) in Lao goats. A total of 1,072 serum samples were collected by convenience sampling across five provinces in Laos and tested for antibody response to PPRV using a commercially available competitive ELISA. Positive antibody responses were found in 2.2% (95% CI 1.4, 3.2) of the samples. True prevalence calculations indicated a total overall sample prevalence of 1.7% (95% CI 0.9, 2.8). The highest provincial seroprevalences were Xiangkhouang (3.5%, 95% CI 1.6, 6.9) and Xayaboury (2.9% (95% CI 1.3, 5.7). There was no association between antibody response and each of the following factors: location, breed, gender or age. Considering the apparent absence of disease manifestation of PPR in Laos, likely explanations for the antibody positivity could include cross reaction to other Morbilliviruses such as Measles or Canine Distemper, importation of pre‐vaccinated goats, need for test cut‐off re‐evaluation to be region specific, or a subclinical and a less virulent circulating virus. This study highlights that the sampled Lao goat population is highly likely to be naïve to PPRV and therefore at risk of an outbreak, possibly by transboundary incursion of livestock from PPR endemic China. Further work is required in the testing of small ruminants in Laos that may eventually provide evidence for a status of freedom from disease, particularly in support of programs aimed at global PPR eradication.

## 1 INTRODUCTION

Peste des Pestes Ruminants virus (PPR) is the most globally widespread infectious disease of small ruminants and threatens food security, sustainability and the welfare of animals and humans across Africa, the Middle East and Asia (Baron, Diop, Njeumi, Willett, & Bailey, 2017; Kumar et al., 2014; OIE, 2016). PPR virus (PPRV) belongs to the Morbillivirus family, along with Distemper, Rinderpest and Measles viruses (Salami et al., 2014).

Following the global eradication of Rinderpest in 2011, the OIE and FAO have joined target PPR as the next animal disease to eradicate (Lancelot, Lancelot, & De Almeida, 2016; OIE, 2016; OIE, & FAO, 2015). Despite efforts, PPR is emerging in new regions in the world causing significant animal and economic losses. Based on data available up until 2014, the virus is present in 65 countries with an additional 20 countries being classed as “at risk” (Jones et al., 2016). A recent cost‐benefit analysis study concluded that global eradication of PPR would see a return of $74 billion over 15 years (Jones et al., 2016). Over 90% of the world's small ruminant population is located in developing countries, providing nutrition, income from trade in animals and their products, plus improved economic stability and resilience for smallholder farmers (Herrero et al., 2013). Eradication of PPR would likely provide considerable sustainability and welfare benefits to vulnerable communities across Asia, the Middle East and Africa.

Peste des petits ruminants virus has a tropism for epithelial and lymphoid cells (Kumar et al., 2014). Clinical signs in small ruminants typically start with dullness and fever, progressing to mucopurulent oral, ocular and nasal discharge, followed by oral lesions, bronchopneumonia and diarrhoea (Albina et al., 2013; Balamurugan, Hemadri, Gajendragad, Singh, & Rahman, 2014; Kumar et al., 2014). Animals can excrete PPRV prior to the onset of clinical signs (OIE, 2016; Parida et al., 2015), with large quantities of PPRV excreted in discharges from infected animals. However, PPRV is not stable in the environment and requires direct transmission in fluids for infectious spread (OIE, 2016; Parida et al., 2015). The extent of clinical signs, morbidity and mortality can depend on the viral strain, the environment and the immune status of the animal (Parida et al., 2015; Ratta et al., 2016; Santhamani, Singh, & Njeumi, 2016). The virus has a high morbidity and mortality, reaching to 100% and over 90% in naïve herds, respectively (Parida et al., 2015). Mortality occurs between 5 and 10 days of onset of infection, with the few recovering animals developing strong lifelong immunity (OIE, 2016; Parida et al., 2015).

There are four lineages of PPRV which have all circulated in Africa (Banyard et al., 2010; Dhar et al., 2002; Dundon et al., 2014; Muniraju et al., 2016; OIE, 2016). Lineage IV historically only affected Asia, although has spread into Western and central Africa over the last two decades and northern Africa with PPRV described in Morocco 2008 and 2015, Algeria 2010 (OIE, 2016; Parida et al., 2015). PPRV Lineage III has also been described in the Middle East (OIE, 2016). The spread of PPR is considered to be a result of transboundary movement of small ruminants (Dhar et al., 2002; Kumar et al., 2014; Liu et al., 2018), with the rapid trading of small ruminants also contributing to the propagation of outbreaks (Balamurugan, Das et al., 2014).

Goat raising can be a successful low input livestock system for smallholder farmers and is increasing in importance in Laos (Burns et al., 2018; Windsor et al., 2017). Although the goat industry in Laos is the smallest livestock sector, recent increase of mutton prices in China and Vietnam have spiked a “goat boom” in SE Asia, leading industry professionals to estimate that the Lao goat population has more than doubled since the 2011 agricultural census (Burns et al., 2018; Windsor et al., 2017). In that census, 6% of households raised goats (Anonymous, 2012).

There are minimal data on the occurrence of PPR in southeast Asia. China has experienced two major outbreaks of PPR in the last decade, although it was previously free from the disease. The first outbreak occurred in Tibet in 2007. This outbreak was likely caused by importation of goats from neighbouring Pakistan and Tajikstan, resulting in the loss of 30.8% of the local population of small ruminants (Bao et al., 2011; Liu et al., 2018). Stamping out of suspected infected herds, delivery of effective vaccination programs, and implementation of nationwide surveillance strategies, led to eradication of PPR in China by 2010 (Liu et al., 2018). The second outbreak occurred in Xienjuang in 2013‐14. This outbreak spread to 32 other counties, including an outbreak in Yunnan on the northern Lao border, a thoroughfare for trade between the Association of Southeast Asian Nations (ASEAN) countries (Li et al., 2017; Liu et al., 2018; Wu et al., 2016). PPR lineage studies suggest this outbreak was likely due to transboundary movement of animals into China rather than a re‐emergence of the disease from Chinese herds (Wu et al., 2016). Prior to these outbreaks in China, PPR antibodies were discovered in apparently healthy mountainous goats in northern Vietnam, yet any attempts to identify the virus and other investigations have not been reported (Maillard et al., 2008).

To date, there have been no reports of PPR outbreaks in Laos. However, the occurrence of similar endemic diseases and poor veterinary infrastructure may lead to PPR being missed or underdiagnosed. Differential diagnoses for PPR in Laos may include foot and mouth disease virus (FMD) (Nampanya et al., 2013), ovine parapoxvirus induced Contagious Ecthyma (Windsor et al., 2017), Coxiellosis (Burns et al., 2018), Brucellosis (Burns et al., 2018) and intestinal parasites (Windsor et al., 2018). Laos is considered as being “at risk” of PPRV incursion due to: proximity and trade with PPR‐endemic China; it is a landlocked country with “porous” borders enabling livestock trade between ASEAN countries; and the relatively poor veterinary infrastructure with suboptimal capacity to detect and respond to emergency and/or emerging disease outbreaks (Bastiaensen, Kamakawa, & Varas, 2011; Nampanya et al., 2013). In addressing the risk of PPR to Laos to protect the livelihoods of small ruminant smallholder farmers from losses, it is important to investigate if PPR is already present in Laos. Seroprevalence studies of PPR can indicate past exposure to PPRV and demonstrate regions to target in future incidence studies and disease control programs. This study was designed to provide baseline information of PPR exposure in Lao goats.

## 2 MATERIALS AND METHODS

### 2.1 Ethics statement

This study was conducted in compliance with State Acts and National Codes of Practice for Ethical Standards, with animal and human ethics approval obtained from The University of Sydney Ethics Committee (project no.2015/765 and 2014/783, respectively). Verbal consent was obtained from all goat owners prior to collection of samples.

### 2.2 Study sites and sample collection

**Figure 1:**
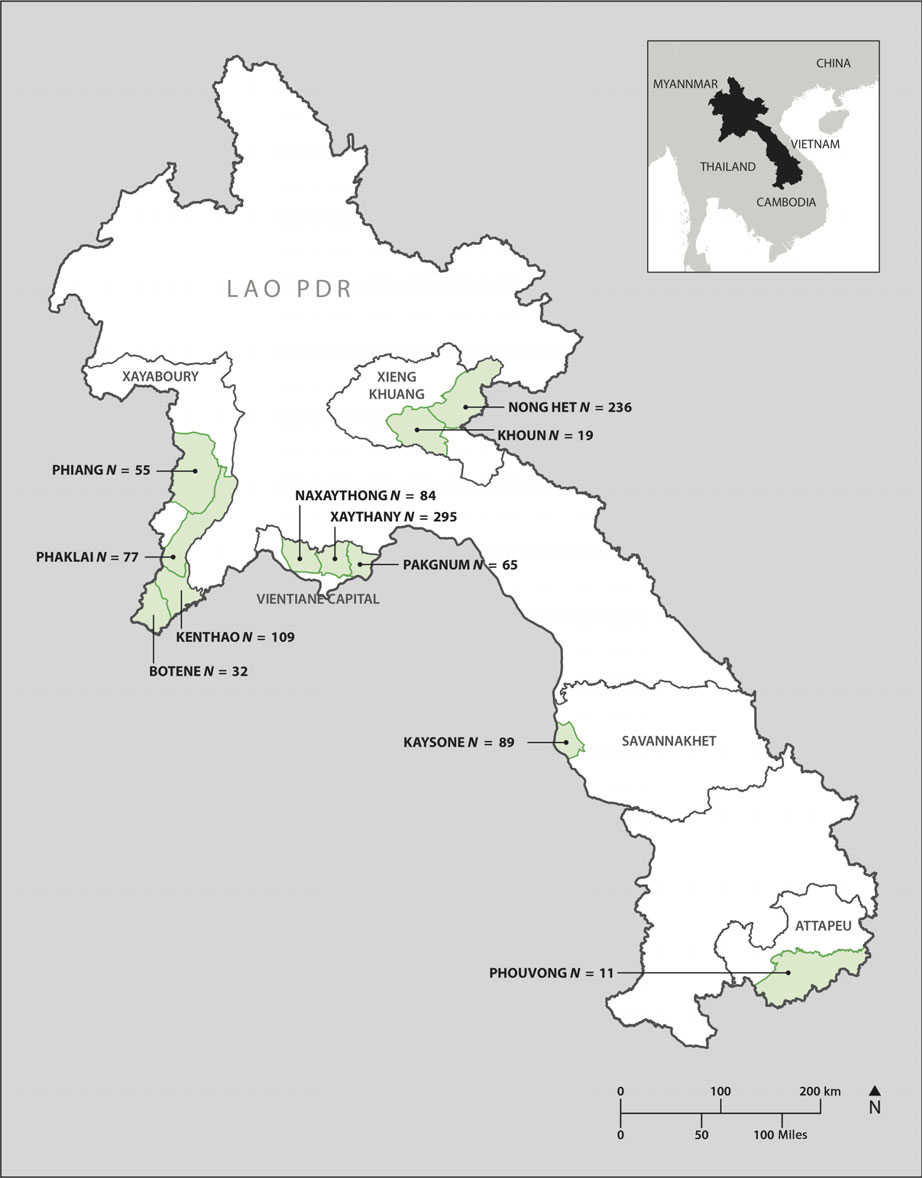
Map of Laos and districts sampled in this study. Numbers of samples collected in each location is indicated [Colour figure can be viewed at http://wileyonlinelibrary.com].

This study was conducted within five provinces in Laos, including Vientiane Capital, Xiangkhouang, Xayaboury, Savannakhet and Attepeu. A total of 1,072 goat sera samples were collected opportunistically during provincial training programs between October 2016 and May 2017 (Figure 1). Samples were originally collected for a recently published study (Burns et al., 2018), with blood collected and sera processed and stored at −80°C until further analysis. Epidemiological data were recorded for all samples, including age via teeth examination and animal gender. Breed information was collected for samples obtained in 2017 only.

### 2.3 Serological testing

Sera was tested for antibodies against PPR using a competitive enzyme‐linked immunosorbent assay (ID‐Screen PPR Competition, product code PPRC‐10P, ID‐Vet, Gabrels, France) according to the manufacturer's instructions (Libeau et al., 1995). Results were expressed as a percentage inhibition of the optical density (%OD) reading of the test sample calculated as % OD = 100 × (S‐N)/(P‐N), with (S) sample and OD of the negative (N) and positive (P) controls, respectively. Samples with % OD < 50 were considered positive. Samples with 50 < OD% < 60 were considered doubtful and were considered negative in this study. Samples with % OD > 60 were considered negative.

### 2.4 Statistical analysis

Data analysis was performed using Stata/SE version 15.0 for Macintosh (StataCorp, College Station, TX). Serological prevalence was calculated as the proportion of animals that had detectable antibodies in the sample population and expressed as a percentage. True prevalence was estimated for overall and spatial groupings using the “AusVet Epitools” website (Ausvet, 2018). The Fischer's exact test was used to determine associations between apparent prevalence and location, breed, age, and gender for samples with epidemiological information available. Associations were considered significant if *p* ≤ 0.05. The 95% Confidence intervals have been reported for the prevalence.

## 3 RESULTS

### 3.1 Overall results

The overall seroprevalence of PPR was 2.2% (23/1,072, 95% CI 1.4, 3.2) (Table 1). Further 2.7% (29/1,072) sera were tested as doubtful. The estimated overall true prevalence was calculated to be 1.7% (95% CI 0.9, 2.8) (Tables 1 and 2). All provinces examined had positive sera (Table 1). Overall, 30.8% (16/52) of villages had at least one seropositive goat (Table 1). Although most had one positive goat per village tested, there were two seropositive goats each in the Xiangkhouang villages of Phalouang, Phapher and Thamthom and the Xayaboury village of Souvanhaphoum (Table 1). There were three positive goats in the Xayaboury village of Ponhin (Table 1) and these were all from the same household (3/7 (42.9%)).

**Table 1:** Antibody response to PPR C‐ELISA in Lao goats by village

Province	District	Village	Number	Positive	Prevalence
Attepeu	Phouvong	Nongvilaynuae	11	1	9.1%
Savannakhet	Kaisone	Dongbang	10	0	0
		Nongkom	22	1	4.6%
		Sorkvang	20	0	0
		Yangphosy	16	0	0
		Unknown name	21	0	0
Vientiane CPT	Naxaythong	Hong Noir	31	1	3.2%
		Nayong	33	1	3.0%
		Sisawan	20	0	0
	Parknguem	Naxon	65	0	0
	Xaythany	Douagbouthdee	70	0	0
		E Lay	10	0	0
		Hai	8	0	0
		Lard Khouey	26	0	0
		Nathae	80	1	1.3%
		Phailorm	48	0	0
		Ponsawanh	27	1	3.7%
		Thongmang	26	0	0
Xayaboury	Boten	Nasan	32	1	3.1%
	Kentao	Donmen	17	0	0
		Hatdeung	41	0	0
		Hoilot	37	0	0
		Meungow	5	1	20.0%
		Nahin	9	0	0
	Paklai	Namsong	8	0	0
		Nasawan	15	0	0
		Sanglai	12	1	8.3%
		Souvanhaphoum	42	2	4.8%
	Phiang	Donguang	3	0	0
		Nampouy	27	0	0
		Ponhin	25	3	12.0%
Xiangkhouang	Khoun	Nampharn	6	0	0
		Nampho	3	0	0
		Sankheeng	8	0	0
		Youn	2	0	0
	Nonghed	Khorkmou	7	0	0
		Kieowpatou	19	1	5.3%
		Korthong	5	0	0
		Longkouang	8	0	0
		Nammen	5	0	0
		Nong‐Aor	21	1	4.8%
		Nonghed‐tai	5	0	0
		Nongkob	13	1	7.7%
		Pahok	20	0	0
		Pakhom	10	0	0
		Phalouang	18	2	11.1%
		Phapher	30	2	6.7%
		Phouhoaxang	8	0	0
		Thamkhou	20	0	0
		Thamthoum	19	2	10.5%
		Yodkhor	5	0	0
		Yordsao	20	0	0
Total	11	52	1,072	23	2.2%

**Table 2:** Antibody response to PPR C‐ELISA in Lao goats

Variable	Region	No.	Positive	Apparent prevalence (95% CI)	True prevalence (95% CI)	Fischer's exact *p*
Province	Attepeu	11	1	9.1% (0.2, 41.3)	9.1% (0.0, 29.6)	
	Savannakhet	89	1	1.1% (0.0, 6.1)	0.6% (0.0, 5.9)	
	Vientiane CPT	444	4	0.9% (0.3, 2.3)	0.3% (0.0, 1.8)	
	Xayaboury	273	8	2.9% (1.3, 5.7)	2.5% (1.0, 5.4)	
	Xiangkhouang	255	9	3.5% (1.6, 6.9)	3.1% (1.4, 6.4)	0.057^a^
Breed	Introduced	297	4	1.4% (0.4, 3.4)	‐	0.556
	Native	340	7	2.1% (0.8, 4.2)	‐	
Sex	Female	849	15	1.8% (0.1, 2.9)	‐	0.175
	Male	211	7	2.1% (1.3, 6.7)	‐	
Age	>3	268	5	1.9% (0.6, 4.3)	‐	1.000
	1–3	267	4	1.5% (0.4, 3.4)	‐	
	>1	267	4	1.5% (0.4, 3.4)	‐	
Total		1,072	23	2.2% (1.4, 3.2)	1.7% (0.9, 2.8)	

a Association test did not include results from Attepeu province due to small sample size.

### 3.2 Serological associations

There was a marginal association between PRR antibody positive goats and province (*p* = 0.057), with Xiangkhouang having an apparent prevalence of 3.5%, Xayaboury 2.9%, Savannakhet 1.1% and Vientiane Capital 0.9% (Tables 1 and 2). The results from Attepeu province were excluded from the association test as the sample size was too small with only 1/11 positive samples (Table 2).

There was no significant association between breed (*p* = 0.556, 637/1,072 (59.4%) samples analysed), gender (*p* = 0.175, 1,060/1,072 samples analysed) and age (*p* = 1.000, 802/1,072 samples analysed) (Table 2).

### 3.3 ELISA results

**Figure 2:**
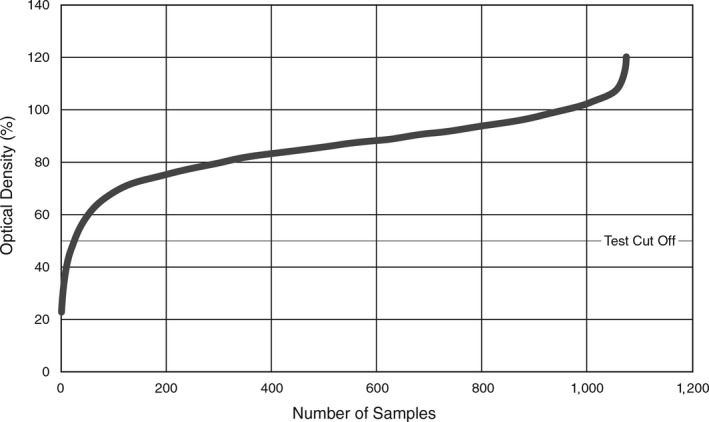
Distribution of ID‐Vet PPR C‐ELISA optical densities for the Lao goat samples tested in this study

The optical density results of each sample including the manufacturer's recommended diagnostic cut off points are demonstrated in Figure 2. The curve demonstrated that the majority of samples giving negative or “normal” results were above 70% inhibition above the lower horizontal asymptote.

## 4 DISCUSSION

Measuring PPR antibody responses in goats in Laos can provide an estimated prevalence status and aid in further targeted studies aimed at providing evidence for a national disease‐free status. This preliminary study provides baseline evidence that there are goats in Laos that have sera that may produce a positive result if tested in a commercial PPR serological assay. However there remains no evidence for the presence of clinical PPR disease in Laos.

This study utilised a commercial competitive ELISA for the detection of PPR antibodies. The development of a competitive ELISA that detected monoclonal antibodies in response to a PPR recombinant nucleoprotein was a breakthrough in mass surveillance of PPR, allowing inexpensive testing of mass samples with rapid result turnaround (Libeau et al., 1995). The test has reported 94.5% sensitivity and 99.4% specificity and has proven to be successful throughout various global surveillance screening programs since 1995 (Lancelot et al., 2016; Libeau et al., 1995; Santhamani et al., 2016). However, the diagnostic cut offs were calculated to have a high specificity for a disparate region and environment to Laos. The diagnostic accuracy study performed 20 years ago may need to be re‐evaluated in a non‐endemic environment to determine a more appropriate diagnostic cut off.

The presence of PPR antibodies in goats can indicate exposure to the virus and potentially, undetected clinical disease. However, without evidence of mass clinical manifestation of PPR throughout Laos, it is considered highly unlikely that the few positive results detected here demonstrate that true exposure to PPR has occurred. Furthermore, as endemically infected countries usually exhibit a considerable number of animals with clinical signs due to the high morbidity and mortality rates from PPRV infection, if infection were present in Laos then much higher numbers of seropositive animals would be expected. The results presented demonstrate lower numbers of seropositive animals in comparison to other unvaccinated goat populations in PPR endemic countries, including Saint Martin's Island, Bangladesh (37.5%) (Siddiqui et al., 2014), Tanzania (34%: 2014 outbreak, 5.6%, 2015) (Lembo et al., 2013), Northern India (11.63%) (Balamurugan, Das, et al., 2014) and Bangladesh (8.7%) (Islam et al., 2016). In this study, only 1–3 goats were seropositive in each village and were distributed throughout the sample population rather than clustered in one district. In an endemic country, the serological results would very likely be clustered. It remains possible that there has been outbreak of PPR in Laos without detection or declaration due to suboptimal veterinary capacity in some areas (Bastiaensen et al., 2011), or that PPR may have been mistaken for other diseases, such as FMD and endoparasites; both are endemic in Laos (Nampanya et al., 2013; Windsor et al., 2018). However, it is also possible, although unlikely, that subclinical disease or mild infections could be responsible for an anti‐PPR antibody response without clinical signs (Balamurugan, Das et al., 2014). Further studies are needed to demonstrate circulation of PPRV in animals suspected of displaying the disease, based on evident clinical signs and demonstration of the infectious agent via real time polymerase chain reaction (RT‐PCR) of infected goat samples or in vitro isolation (OIE, & FAO, 2015).

The presence of antibodies against PPR is not always indicative of previous infection (OIE, 2016). Currently, there is no antibody test that facilitates differentiation of infected and vaccinated animals (DIVA) for PPR (Albina et al., 2013; Libeau et al., 1995; Santhamani et al., 2016). Goats could have been vaccinated prior to transboundary relocation from neighbouring PPR endemic countries such as China or Bangladesh (through Myanmar). It is extremely unlikely that goats could be vaccinated against PPR in Laos as the vaccine has not been made available and is only produced where PPR outbreaks occur. It is possible that the few serological reactors in this study could have been caused by an immune response of the goats to another morbillivirus, such as distemper or measles and close proximity to infected dogs or humans, respectively, causing an antibody response with the absence of clinical disease or classical disease response. There is strong evidence demonstrating that cross species infection and immune responses of the morbilliviruses occurs (Beineke, Baumgärtner, & Wohlsein, 2015; Cosby & Weir, 2018; Ratta et al., 2016; Sakai et al., 2013; Sheshberadaran, Norrby, McCullough, Carpenter, & Orvell, 1986). Specifically, measles is still prevalent in parts of Laos (World Health Organisation, 2016) and canine distemper virus is anecdotally causing active infection in Laos and is present in neighbouring Thailand (Techangamsuwan et al., 2015). There is currently no ability to differentiate a cross reaction from a true PPR antibody reaction and this interferes with serological surveys when regions are required to prove freedom from disease for global PPR eradication (Santhamani et al., 2016).

While this study cannot determine if there is active circulation of PPRV throughout Laos, it does indicate that, the goat population in Laos is largely naïve to the virus and therefore at risk of outbreak. The main risk of PPRV to Laos is movement of goats across borders (Balamurugan, Hemadri, et al., 2014; Dhar et al., 2002; Kumar et al., 2014). The outbreak in China during 2013‐14 affected 256 counties including Yunnan, a border province with Laos (Li et al., 2017; Liu et al., 2018; Wu et al., 2016). It is possible that the lack of border security plus illegal movement of animals between countries in the region may spread PPR into Laos and beyond into other ASEAN countries, such as Thailand, Vietnam, Cambodia and Malaysia. Wildlife crossing borders may also play a role in the spread of PPR into Laos (Li et al., 2017). Although the National Animal Health Laboratory is capable of providing adequate diagnostic support, veterinary work in Laos relies largely on minimally trained provincial veterinary teams and untrained village veterinary workers and the sector is underfunded, with low capacity to respond in an emergency disease outbreak situation (Bastiaensen et al., 2011).

It is interesting that the provinces with the highest seropositivity were Xiangkhouang, located close to Chinese and Vietnamese livestock trade, and Xayaboury, a thoroughfare for traffic from Myanmar (neighbouring Bangladesh) and Thailand into Laos then continuing to China. As these were the only provinces that had villages with more than one PPR seropositive goats, it is possible that goats located here could have travelled with pre‐exposure to PPR in endemic China or Bangladesh respectively, therefore displaying antibody responses without circulating the virus in Laos. Future investigations of PPR in Laos should target these areas. Although the sample size was too small to determine if there were significant correlations between districts and villages and seroprevalence, the study demonstrated that there was no correlation between gender, age and breed and seroprevalence (Woma et al., 2016). In other studies in PPR endemic countries, older animals are correlated with antibody response due to more opportunity of exposure and also likelihood of surviving an outbreak (Abubakar, Javed Arshed, Hussain, & Ali, 2011). Of interest is that in a study performed prior to the Chinese outbreak of PPR in 2008, there was a 2% seroprevalence of PPR antibodies in mountain goats in Vietnam on the border of China, with lack of clinical manifestation of PPR (Maillard et al., 2008). The authors speculated that co‐evolution caused host immunity for the goats and loss of virulence for the virus leading to an antibody response in the absence of clinical signs (Maillard et al., 2008). Further molecular, immunogenic and virulence studies are required to evaluate if this speculation is true for Laos and Vietnam.

A limitation of this study is that for reasons of necessity it employed convenience sampling rather than randomised sampling. This may have skewed the data to higher socioeconomic farms with greater access to roads, although considering that PPR is mainly transmitted by movement of animals, this would likely have overestimated the seroprevalence. True prevalence was calculated to account for sensitivity and specificity parameters of the test rather than to declare a “true prevalence” in these sample areas as convenience sampling is not representative of the population as a whole.

Further work is needed to ensure that Laos can continue to participate in the global eradication of PPR. The following is an outline of future targets:

- Future studies. It is clear that targeted monitoring for the presence of disease should occur in Xayaboury and Xiangkhouang, northern provinces bordering China such as Phongsali and Luangnamtha and also major road thoroughfares throughout Laos. This monitoring should include recording clinical signs, RT‐PCR testing, serology testing and risk assessment (OIE & FAO, 2015). Participatory epidemiology will be a key in understanding if there have been any outbreaks or clinical signs in the past in these at risk areas (OIE, & FAO, 2015).
- Capacity building. For future incidence studies to be successful, capacity building workshops including education and training of provincial, district, veterinary and border security staff on recognising and understanding PPR is essential. PPR virions are excreted for 10 days post onset of fever: hence incidence studies require quick sampling of suspect animals (Parida et al., 2015). This is achievable as demonstrated through widespread knowledge of FMD virus throughout the country (Nampanya et al., 2016). Improving the capacity of regional laboratories will be beneficial for a more rapid diagnosis and potentially disease management response to a PPRV outbreak, plus increased diagnostic abilities for additional livestock diseases.
- Political and financial support. For Laos to participate in the eradication of PPR, the virus needs to become a notifiable disease with incentives provided for villages to report in Laos. An Emergency Animal Disease Plan needs to be prepared for a possible outbreak response, including policies on stamping out, vaccination and movement restriction considerations (OIE, & FAO, 2015). In June 2017, China hosted an outbreak investigation conference and invited staff members from ASEAN national laboratories, including training on disease recognition and RT‐PCR diagnosis of PPR. This regional collaboration is crucial for global eradication of PPRV. Finally, there will need to be significant funding from national governments and external bodies for Laos to participate in global eradication.

The growing goat industry in Laos is at risk of PPRV. The few antibody responses detected in this study are more likely to represent cross reactions or the need for test refinement, rather than evidence of circulating virus in the absence of reported clinical signs. Further work is needed for Laos to participate in the global eradication of PPRV including targeted incidence studies, capacity building and political support to ensure that the Lao veterinary sector can respond in a PPR crisis.

## CONFLICT OF INTEREST

All authors declare that they do not have a conflict of interest.

## DISCLAIMER

Material has been reviewed by the Walter Reed Army Institute of Research. There is no objection to its publication. The opinions or assertions contained herein are the private views of the authors, and are not to be construed as official, or as reflecting true views of the Department of the Army or the Department of Defence.
